# Vice Versa

**Published:** 1869-11

**Authors:** 


### VICE VERSA.

THE "Dental Office and Laboratory," in a complimentary notice of the Dental Register, gets the editors' names reversed from their usual order. This comes from straining his eyes recently in looking at an *os coccygis*, which had wandered away and hid itself in the front of the subject's thigh. Rest and goggles! Mr. Editor. W.

